# Relapse in Eating Disorders: A Systematic Review and Thematic Synthesis of Individuals' Experiences

**DOI:** 10.1002/cpp.70101

**Published:** 2025-07-01

**Authors:** Natasha Heal‐Cohen, Sophie M. Allan, Nieve Gauvain, Rachel Nabirinde, Aaron Burgess

**Affiliations:** ^1^ Cambridgeshire and Peterborough NHS Foundation Trust; ^2^ Department of Clinical Psychology and Psychological Therapies, Norwich Medical School University of East Anglia Norwich UK; ^3^ School of Health Sciences University of East Anglia Norwich UK; ^4^ Norfolk & Norwich University Hospital Norwich

**Keywords:** eating disorder, qualitative, recurrence, relapse, systematic review

## Abstract

**Objective:**

Relapse is common in eating disorders (EDs); however, it has received significantly more attention within quantitative research. This systematic review aimed to synthesize qualitative findings regarding the experiences of relapse for people with EDs.

**Method:**

A search for studies reporting qualitative data that included experiences of relapse in individuals with EDs was conducted. This included a systematic search of MEDLINE, CINAHL, PsycInfo, Scopus, SSCI, and PQDT Global along with supplementary searching. A total of 1594 titles and abstracts and 168 full texts were screened for eligibility. Sixteen studies were included in the review. Quality appraisal was conducted using the CASP checklist. Data were extracted from each paper and thematic synthesis of relevant data from study findings/discussion was completed in NVivo.

**Results:**

Most included studies involved female participants in the United States, Canada, and United Kingdom with anorexia and bulimia nervosa. Five analytical themes were generated: 1) “I wasn't letting go”: Relapse as enticing; 2) “Bound to lose”: Relapse as unstoppable; 3) “If the going gets tough I've always got this”: Relapse as protective; 4) “Coming back with your tail between your legs”: Relapse as destructive; 5) “So much of this journey … is learning”: Relapse as instructive.

**Discussion:**

Findings highlight the gap between psychological and behavioral improvements that precede relapse, and the contrasting ways relapse is described and experienced. They support a focus on motivational factors and underlying psychological difficulties in treatment, extending beyond a behavioral focus. Further research is needed to understand relapse experiences among males and individuals from the global majority.

**Summary:**

- Individuals with EDs who relapsed described desiring or feeling powerless to control relapse. Findings suggest the need for greater emphasis on developing intrinsic motivation and self‐efficacy in order to sustain recovery.
- Residual ED cognitions and unaddressed psychological vulnerabilities were implicated in relapse. Findings caution against premature discharge before sufficient cognitive and emotional progress has been made.
- In response to relapse, clinicians should aim to foster hope, compassion and tailor treatment to address the recovery needs revealed by relapse.

Relapse, broadly defined as “the deterioration in a patient's condition after a partial or apparently complete recovery” (Oxford English Dictionary 2024), is common among individuals with EDs. There is no standardized clinical definition of relapse among EDs, and research has operationalized it in varied ways, including a return to meeting diagnostic criteria, re‐hospitalization, and more often focus on changes in physical health and behaviors (e.g., weight, frequency of bingeing and purging) rather than psychological indicators (de Rijk et al. 2024; Miskovic‐Wheatley et al. 2023). Whilst definitions influence reported rates of relapse, a recent meta‐analysis revealed that 26% of individuals with EDs globally experienced relapse after recovery (Solmi et al. 2024). However, as most included studies were conducted in North America and Europe further research from other regions is needed to gain a more comprehensive understanding of relapse patterns worldwide.

Relapse prevention has been a key focus within support for EDs, typically addressed in the final stages of treatment and increasingly in post‐treatment interventions (Robinson et al. 2006; Schlam and Wilson 2007; Steinglass et al. 2022). However, The National Institute for Health and Care Excellence (2017) highlight the lack of evidence of how to effectively prevent relapse in anorexia nervosa (AN), listing the topic as a research priority. What is more, aspects of treatment have been perceived as contributing towards “revolving door” experiences (Joyce et al. 2019) in which short‐lived improvements lead to an in‐and‐out cycle of accessing care. Understanding relapse is essential in informing support to both prevent and better meet the needs of those who relapse.

Current research indicates that individuals are particularly vulnerable to relapse in the first year after treatment (Berends et al. 2018; Nagl et al. 2016). Relapse is also more likely among those still experiencing ED symptoms (i.e., partial remission); however, it remains a significant risk even after full recovery (Khalsa et al. 2017). Varied predictors of relapse in EDs, primarily AN, have been identified, including weight and shape concerns, comorbidities such as depression, body mass index at discharge, stressful life events, and psychosocial functioning (Berends et al. 2018; de Rijk et al. 2024; Grilo et al. 2012; Keel et al. 2005; Sala et al. 2023). However, current quantitative research is limited in its ability to capture the complex dynamic processes involved in relapse (Berends et al. 2018; Pars et al. 2024).

Individuals who relapse may have received treatment, developed knowledge and skills, and shown motivation and efforts towards recovery. How relapse can occur within this context, and how individuals experience this, is important to understand. Models of relapse, often developed in the context of addiction, have been applied to EDs (Schlam and Wilson 2007). The transtheoretical model of change (TMC; Prochaska and DiClemente 1992) sees change as a cyclical process where relapse triggers a recycling through earlier stages of change during which learning occurs. Freeman and Dolan's (2001) expansion of the model described stages of pre‐lapse, characterized by thoughts and desires for the “old” times, and lapse, which involves a decrease in skills needed to maintain recovery and a return to old patterns of thinking, and finally relapse, in which individuals return to old behaviors to a greater or lesser extent. Marlatt and Gordon's (1985) cognitive behavioral model conceptualizes relapse as a process: a series of events unfolding over time, with individuals' appraisals and responses being central to this process. More recent revisions emphasize the dynamic interactions between proximal and distal factors underlying relapse (Witkiewitz and Marlatt 2007).

Qualitative research enables nuance, depth, and complexity of exploration, highlighting experiences unmeasured or immeasurable in quantitative research (Clarke and Braun 2013). It has the potential to capture individuals' appraisals and responses to relapsing considered central to the process (Marlatt and Gordon 1985). Qualitative reviews of EDs during COVID‐19 (Schneider et al. 2023) and the perinatal period (Fogarty et al. 2018) identified and explored relapse during these periods of upheaval. Outside of these contexts, relapse has received little qualitative examination among reviews focused on treatment and recovery (Gustafsson et al. 2021). To our knowledge, no systematic review has specifically focused on the experience of relapse for people with EDs despite its prevalence and clinical significance. This seems an important complement to recent quantitative syntheses (de Rijk et al. 2024; Sala et al. 2023; Solmi et al. 2024).

## 1 Aims

This thematic synthesis of qualitative studies seeks to deepen understandings of the experiences of relapse for people with EDs. It aims to generate insights that can inform theories of ED relapse as well as policy and practice around relapse prevention and treatment. As such, the review question was: *What are the experiences of people with an eating disorder (ED) who encounter relapse(s)?*

## 1 Methods

The review was prospectively registered with PROSPERO (CRD42023492922). It has been reported according to PRISMA (Page et al. 2021) and ENTREQ guidelines (Tong et al. 2012).

### 1.1 Design

The theoretical framework for this review is critical realism. As such, it assumes an underlying shared reality while recognizing that personal and social contexts shape how relapse is understood. This review is therefore interested in both commonalities and the diversity and nuance in how relapse is described, interpreted, and experienced. A context‐mechanism‐outcome (CMO) framework (Pawson and Tilley 1997) acted as a sensitizing framework, providing a loose structure to help orientate towards processes related to relapse which was drawn upon through various stages of the review.

### 1.2 Definition and Scope

In line with the critical realist framework, in the absence of standardized definitions this review adopted a broad working definition of relapse as a resumption or worsening of ED symptoms following a period of improvement (e.g., partial or full remission or recovery). No specific parameters were set around the duration or extent of prior recovery or subsequent deterioration in determining relapse. Within the review, relapse was identified based on i) references to relapse and descriptions aligning with the working definition, ii) re‐entry into treatment, and iii) relapse definitions provided by included study authors.

The operationalization of experiences of relapse was guided by a CMO framework. Within this, experiences related to perceived triggers and warning signs of relapse, thoughts and feelings about relapse and initial responses to relapse (including help seeking) were all of interest. Experiences of treatment and recovery from relapse, or successfully preventing relapse were beyond the scope of the review.

### 1.3 Eligibility

Inclusion criteria were as follows: (1) study design—primary qualitative studies or mixed‐methods studies which separately analyze and report qualitative results; (2) language—written in English; (3) population—studies involving individuals who are experiencing or have experienced anorexia nervosa (AN), bulimia nervosa (BN), binge eating disorder (BED), other specified feeding and eating disorder (OSFED) or the replaced diagnosis of eating disorder not otherwise specified (EDNOS) in line with the theoretical and empirical support for the transdiagnostic conceptualization of these disorders (Fairburn et al. 2003; Gordon et al. 2018); and (4) focus—studies in which these individuals' experiences of ED relapse, as operationalized in *Definition and scope*, are a key theme within results/discussion.

Exclusion criteria were as follows: (1) paper type—single case studies, book chapters, book reviews, opinion pieces, conference presentations, posters and meeting abstracts; (2) population—studies where individuals without the specified disorders comprised 20% or more of participants unless eligible participants were separately analyzed and reported; (3) focus—studies where relapse is discussed exclusively with respect to preventing relapse or to treatment for/recovery from relapse; and (4) context—studies where relapse is discussed exclusively in the context of the perinatal period or COVID‐19. Pilot searches revealed a disproportionate number of papers in these areas, and therefore their inclusion was deemed to risk shifting the review away from its intended focus on relapse beyond these specific contexts which have received their own examination in recent reviews (Fogarty et al. 2018; Schneider et al. 2023).

### 1.4 Search Process

A comprehensive systematic search strategy was conducted following consultation with an academic librarian. Six electronic databases were searched: MEDLINE (EBSCO host), APA PsycInfo (EBSCO host), CINAHL Ultimate (EBSCO host), Social Sciences Citation Index, Scopus, and ProQuest Dissertations & Theses Global (PQDT Global). Databases aimed to cover medical, psychology and social sciences fields, with Scopus offering additional breadth and PQDT Global identifying unpublished academic papers which can be important, otherwise overlooked sources of rich material. All years were included from inception through to the initial search date in January 2024. Follow‐up searches were then conducted, with the final search in November 2024.

The SPIDER tool (Sample, Phenomenon of Interest, Design, Evaluation, Research type) was used to inform and standardize the electronic search strategy (Cooke et al. 2012). Terms related to “eating disorders” (Sample), “relapse” (Phenomenon of Interest), and “qualitative research” (Research Type) were combined using the Boolean operator AND. Terms were searched within title, abstract and keyword fields (or closest database equivalent) and index terms were included where available (see Supplement S1 for full search strategy).

To supplement this, related qualitative ED reviews and reference lists of included studies were hand searched, and leading researchers in the field were contacted to identify additional studies.

**FIGURE 1:**
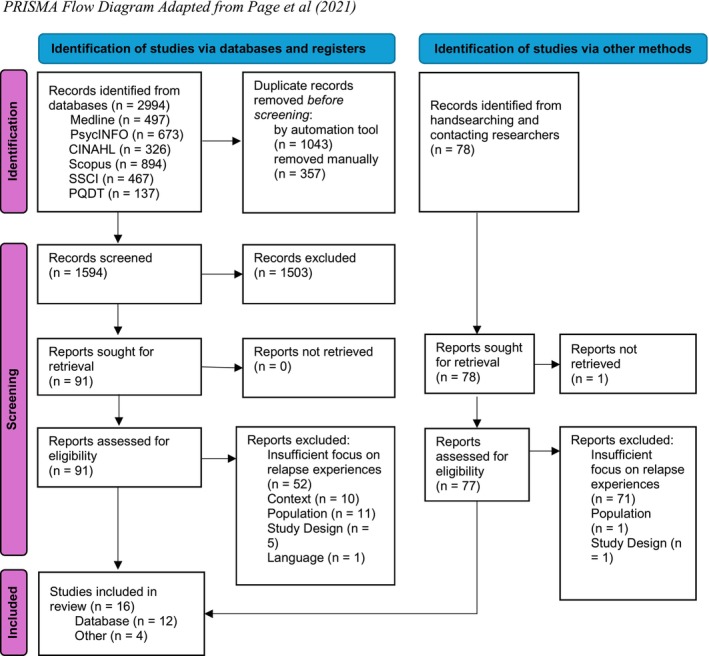
Flow diagram adapted from Page et al. (2021).

Figure 1 shows a PRISMA flow diagram of the screening process. Database searches yielded 2994 records (2796 in initial search and 198 in follow‐up searches) which were exported to Endnote for deduplication. All screening was completed by NHC in research platform Rayyan with 20% of titles and abstracts also independently screened by RN and 25% of full texts also independently screened by RN and NG. Any discrepancies were resolved through discussion.

After deduplication, 1594 titles and abstracts were screened, resulting in 91 full texts retrieved and assessed for eligibility from database searching and 77 identified through supplementary searches. SA and AB provided an additional check on final inclusion decisions, reviewing 50% of included studies and a sample of excluded studies to verify the application of eligibility criteria.

In total, 16 studies met inclusion criteria (14 in initial search and two in follow‐up searches). Most excluded studies had minimal content on relapse, for example, a few sentences or one quote (e.g., Offord et al. 2006) or did not distinguish relapse from broader challenges in recovery (e.g., Arthur‐Cameselle and Quatromoni 2014). A handful of papers with a key theme on relapse were excluded due to a lack of information on participants' EDs (e.g., Musolino et al. 2018). One paper was excluded because there was no full text available in English (see Supplement S2).

Data from included studies were extracted by NHC into a Microsoft Excel sheet. Extracted data included study aims, country, setting, sample demographics, illness information, data collection, and analysis methods. Select authors were contacted for clarification of their sample. Full texts were imported into computer software NVivo (version 20) for synthesis.

### 1.5 Critical Appraisal

The Critical Appraisal Skills Programme Qualitative Checklist (CASP; https://casp‐uk.net/casp‐checklists/CASP‐checklist‐qualitative‐2024.pdf) was used to assess study quality, given its wide use, accessibility, and endorsement (Noyes et al. 2018). Specific adaptations recommended by Long et al. (2020) were adopted to improve its value. The checklist covers indicators of quality throughout the research process, from the appropriateness of study aims to the value of findings. Papers were rated against each checklist item (no, cannot tell, somewhat, yes), with the addition of *somewhat* to indicate the paper partially satisfied the criteria.

Papers were given an overall quality rating (high, medium, low) informed by the checklist with greater weight afforded to analytical rigor given the importance of this for review credibility. The value of the study for the current review was also appraised (very high, high, medium, low), informed by the extent and depth to which it addressed experiences of relapse. NHC and NG independently rated all studies against each criterion and differences in ratings were resolved through discussion.

Quality ratings did not impact study inclusion due to the potential to adversely impact review generalizability (Garside 2014) but informed sensitivity analyses. Post‐synthesis qualitative sensitivity analyses recommended by Carroll and Booth (2015) examined the relative contribution of studies of different quality to the review using NVivo coding data.

### 1.6 Thematic Synthesis

The method of thematic synthesis, described by Thomas and Harden (2008), was utilized for this review. The approach, underpinned by critical realism, is well suited to preserving uniqueness and commonalities of experience, and capturing a shared reality that can inform policy and practice (Barnett‐Page and Thomas 2009). Unlike other methods such as meta‐ethnography, it can handle both the thin and thick description anticipated in included studies (Tong et al. 2012). It provides a formal transparent method specifically developed for systematic reviews, aiding review quality.

Using NVivo, NHC conducted a line‐by‐line coding of all content in the results and discussion sections of each included paper that pertained to experiences of ED relapse. Discussions were held with SA to review and agree upon the content that was coded. Text could be assigned one or more codes to capture its meaning.

As the CMO framework shaped the operationalization of experience, data broadly pertained to context, mechanisms, and outcomes of relapse. This orientation to the data assisted the organization of codes into inductively generated descriptive themes which were further interpreted to develop analytical themes. Each analytical theme was grounded in the data but aimed to extend beyond mere description (Tong et al. 2012) to capture a core concept about how individuals made sense of relapse.

The generation of themes involved an iterative process, moving back and forth between themes, codes, and the underlying data. To preserve study context in the analysis, coded data were annotated with a summary of study context and synthesis findings were examined to determine whether they could be attributed to particular contexts, such as inpatient settings. All stages of the synthesis were performed by NHC, and codes and themes were discussed, reviewed and refined together with SA and NG. As a robustness check, the thematic synthesis was audited by an independent researcher, resulting in refinements that strengthened the coherence and transparency of the analysis. For example, certain theme and code labels were refined to better specify their boundaries and focus on relapse context versus outcomes.

### 1.7 Reflexivity

The researchers played an active role in constructing this review. The choice of research question was influenced by both professional and personal experiences which provide different and complementary lenses with which to view participants' experiences. While the breadth of the review question and framework aimed to be non‐constraining, awareness of CBT models outlined in the introduction and NHC's clinical training in CBT, likely informed the organization of data (e.g., drawing out interpersonal vs intrapersonal distinctions). The ways in which treatment was implicated in relapse may have been more salient due to NHC's background in evaluation research and the felt sense of frustration it generated. NHC kept a reflective journal throughout the research process, and impressions were discussed within the research team to enhance awareness of subjectivity. NHC's and AB's work across CAMHS inpatient and community ED services, helped to guide the translation of findings into clinical implications.

## 2 Results

### 2.1 Overview of Studies

Sixteen studies were included in the review and Table 1 gives an overview of their characteristics. Papers constituted 11 journal articles, and five academic theses published between 1998 to 2024, with more papers concentrated in the latter half of this period. Studies were conducted in the United States (5), United Kingdom (5), Canada (3), Sweden (1), Finland (1), and China (1). They reported on the experiences of 342 participants (98.0% female) with EDs including BN (37.5%), AN (36.3%), both AN and BN (8.8%), and BED (5.3%). Most studies were recruited from ED treatment settings. The extent of participants' recovery prior to relapse varied, as did the severity of symptom return in relapse, ranging from an episode of bingeing or purging in recovery to symptoms severe enough to require hospitalization. Participants ranged from those currently experiencing a relapse to those who had sustained recovery from their last relapse for many years.

**TABLE 1:** Overview of included study characteristics.

Study, country, paper type	Study aim	Participant demographics	Illness information	Recruitment	Data collection and analysis methods	Value for review	Quality^a^	Review themes^b^
Bell et al. (2024), United Kingdom, Journal	To explore how individuals with AN experience self‐disgust as they recover from their ED	*N* (*n* relapsed) = 12 (NR) 100% F Age (mean) = 19–36 (26) Ethnicity = White or White British: 11, British: 1 SES (education) = tertiary education: 4, undergraduate degree: 4, postgraduate degree: 4	100% AN (33% AN‐BP) Duration = NR Relapse^c^ = working def. Recovery status^d^ = physically recovered and subclinical symptom levels	Research participation schemes and platforms and Facebook support groups	Semi‐structured interviews; interpretative phenomenological analysis	Medium	Medium	1–4
Botham (2019), United Kingdom, thesis	To explore through narrative inquiry methodology the experiences of four participants as they navigated their lives away from SE‐AN	*N* (*n* relapsed) = 4 (4) 100% F Age = NR Ethnicity = NR SES (employment) = part‐time: 2, full time: 1, NR: 1	100% AN (AN‐R and AN‐BP) Duration = all > 10 years Relapse = working def. and return to treatment Recovery status = “healthy recovery” achieved	Via professional contacts	Unstructured, open‐ended interviews; narrative analysis	High	High	1–5
Cockell et al. (2004), Canada, journal	To identify factors that help or hinder the maintenance of change and the ongoing promotion of recovery during the critical 6 months immediately following ED treatment	*N* (*n* relapsed) = 32 (6) 100% F Age (mean) = (27.9) Ethnicity = NR SES = upper middle class (Hollingshead's 1975 index = 2.0, SD = 1.03)	AN = 21, EDNOS = 11 Duration = mean 11.6 years Relapse = return to meeting DSM‐IV criteria for AN or BN 6 months after discharge from residential treatment and partial recovery Recovery status = relapse ongoing	Residential treatment program	Interviews; grounded theory	High	Medium	1–4
De Barbieri (2005), United States, thesis	To identify the types of learning that adult women with BN reported as significant to their recovery and the factors that facilitate or impede learning	*N* (*n* relapsed) = 24 (NR) 100% F Age (mean) = 19–58 (NR) Ethnicity = Northern European: 9, Native American: 2, Eastern European: 2, Mediterranean: 2, Other: 2 SES (annual household income) = > $75 K: 8, $50‐75 k: 6, $15–40: 2, < $15 K: 4	100% BN Duration = 2‐30 years Relapse = working def. Recovery status = 15 “recovering,” 9 behavioral symptom free for 6+ months	Outpatient treatment facility	In‐depth interviews; multiple case study interpretive approach using inductive and deductive thematic coding	Medium	High	2–5
Federici and Kaplan (2008), Canada, journal	To investigate patients' view of relapse and recovery and their ability and desire to maintain change	*N* (*n* relapsed) = 15 (8) 100% F Age (mean) = (26) Race = 100% Caucasian SES = “middle to upper class”	100% AN (33% AN‐BP) Duration = NR Relapse = weight relapsed BMI < 17.5 on average 14 months after weight‐restored at discharge from intensive inpatient or outpatient treatment and relapse prevention focused CBT Recovery status = relapse ongoing	Treatment setting	Semi‐structured interviews; phenomenological approach	Very high	High	1–5
Keski‐Rahkonen and Tozzi (2005), Finland, journal	To understand the process of recovery for individuals with EDs through their own words	*N* (*n* relapsed) = 158 (NR) 98% F, 2% M Age (median) = 13–53 (21) Ethnicity = NR SES = NR	BN = 52, AN = 32, AN and BN = 29, BED = 12, orthorexia = 2, NR = 28 Duration = NR Relapse = transtheoretical model of change definition (returning to the problem behavior) Recovery status = NR	Online ED forum	Messages posted by ED forum participants during a 3‐month period; software‐aided text analysis and qualitative methods incl constant comparative method	Medium	Medium	1–5
Liu et al. (2024), United States, journal	To explore what post‐treatment factors patients believe contributed to deterioration, explore post‐treatment skill use and identify motivators and barriers to this	*N* (*n* relapsed) = 12 (11) 75% F, 25% M Age (mean) = 25–65 (40) Race = White: 7, Mixed Race: 2, Asian: 1, Black: 1, Other: 1 SES = NR	100% BN Duration = NR Relapse = 50% increase in symptoms within 34–49 months after CBT‐E treatment and meaningful improvements Recovery status = relapse ongoing for 9, 2 in recovery.	Previous clinical trial of CBT‐E	Qualitative interviews; thematic analysis	High	Medium	1–4
O'Connell (2023), United Kingdom, journal	To examine the author's experience of the diagnosis and treatment of AN	*N* (*n* relapsed) = 1 (1) 100% F Age = NR Ethnicity = NR SES = NR	100% AN‐BP Duration = NR Relapse = working def. and return to inpatient treatment Recovery status = recovered for 7 years	N/A	Author's retrospective account, diary entries and medical records; autoethnography	High	Medium	1,2,4,5
Pilote (1998), Canada, thesis	To describe the stressors encountered by female adolescents during a relapse of BN	*N* (*n* relapsed) = 3 (3) 100% F Age (mean) = 16–19 (NR) Ethnicity = NR SES = NR	AN‐ > BN = 1, BN = 2 (DSM‐IV) Duration = NR Relapse = meeting DSM‐IV criteria for BN after 12 weeks+ of treatment at ED clinic and behavioral improvement Recovery status = in treatment for relapse	ED clinic	Dialogical semi‐structured interviews; descriptive phenomenological method	Very high	Medium	1–5
Seed et al. (2016), United Kingdom, journal	To explore the experience of detention under the Mental Health Act for AN and how this impacts on recovery	*N* (*n* relapsed) = 12 (11) 100% F Age (mean) = 18–43 (NR) Ethnicity = NR SES (employment) = part‐time: 5, unemployed: 5, home‐duties: 1, NR: 1	100% AN Duration = NR Relapse = working def. and return to treatment following start of physical and cognitive recovery under involuntary inpatient admission Recovery status = 11 with current AN symptoms (4 inpatient), 1 in sustained recovery	Treatment settings and ED charity	Interviews; grounded theory	Medium	High	1–4
Stockford et al. (2018), United Kingdom, journal	To explore the general experiences of women with SE‐AN as well as their experiences regarding their treatment.	*N* (*n* relapsed) = 6 (6) 100% F Age (mean) = 33–48 (36) Ethnicity = NR SES = NR	100% AN Duration = 14–28 years (mean 21 years) Relapse = working def. and return to treatment following variety of clinical interventions Recovery status = current AN symptoms	ED service	Semi‐structured interviews; interpretative phenomenological analysis	Medium	High	1–4
Strand et al. (2017), Sweden, journal	To explore patients' experiences of self‐admission to an inpatient ward	*N* (*n* relapsed) = 16 (16) 94% F, 6% M Age (mean) = 18–56 (31) Ethnicity = NR SES = NR	100% AN Duration = 3–42 years (mean 15 years) Relapse = working def. and self‐admitted return to inpatient treatment Recovery status = current AN or AN in partial remission	ED center	Semi‐structured interviews; qualitative content analysis	Medium	High	1,2,4,5
Tibbits (2019), United States, thesis	To explore how women who relapse from BN or BED perceive what factors led to relapse and then recovery	*N* (*n* relapsed) = 12 (12) 100% F Age (mean) = 23–43 (NR) Ethnicity/Race = White or Caucasian: 7, Hispanic: 2, Latina: 1, South Asian American: 1, Biracial: 1 SES = NR	BN = 6, BED = 6 Duration = NR Relapse = working def. (participant‐defined relapses ranged from one bingeing episode to many months in duration) Recovery status = in recovery for 6 + months	Facebook support groups	Semi‐structured Interviews; feminist phenomenological analysis	Very high	High	2–5
Warchol (2013), United States, thesis	To gain knowledge about, understand, and describe the experiences of social comparison within residential treatment facilities from the perspectives of patients diagnosed with BN	*N* (*n* relapsed) = 5 (4) 100% F Age (mean) = 20–31 (23.4) Ethnicity = European American: 4, mixed European American and Asian American: 1 SES (employment) = employed: 3, unemployed: 2	100% BN Duration = NR Relapse = working def. and return to treatment Recovery status = in residential treatment for relapse	Residential ED treatment facility	Semi‐structured interviews; phenomenological analysis	Medium	High	1–5
Wasson (2003), United States, journal	To describe the relapse experiences of women with BN through a qualitative analysis of their experiential accounts	*N* (*n* relapsed) = 26 (26) 100% F Age (mean) = 20–59 (NR) Race = 100% Caucasian SES = NR	100% BN purging type (DSM‐IV) Duration = NR Relapse = bingeing or purging episodes whilst in recovery Recovery status = behavioral symptom free for 6 months+	Overeaters anonymous	Focus groups and individual interviews; qualitative grounded theory techniques and constant comparison method	High	Medium	1–5
Wu and Harrison (2019), China, journal	To understand the experiences of four adolescents receiving inpatient treatment for EDs in China.	*N* (*n* relapsed) = 4 (3) 100% F Age (mean) = 16–19 (NR) Ethnicity = Chinese SES = NR	100% AN‐BP Duration = mean 3.7 years Relapse = working def. and return to inpatient treatment following physical and cognitive improvements in treatment Recovery status = currently inpatient	Online forum	Semi‐structured interviews; interpretative phenomenological analysis	Medium	High	1–3

Abbreviations: AN, anorexia nervosa; AN‐BP, binge‐purge subtype; AN‐R, restricting subtype; BED, binge‐eating disorder; BMI, body mass index; BN, bulimia nervosa; CBT(‐E), cognitive behavior therapy (enhanced); DSM, Diagnostic and Statistical Manual of Mental Disorders; ED, eating disorder; EDNOS, eating disorder not otherwise specified; F, female; M, male; *N* (*n* relapsed), number of participants (number who have relapsed); NR, not reported; SE‐AN, severe and enduring AN; SES, socio‐economic status.

^a^ See Table S4 for an item‐level breakdown.

^b^ Indicates the themes each study contributes to.

^c^ Indicates how relapse was determined in this study (working def. = a resumption or worsening of ED symptoms following a period of improvement).

^d^ Indicates the status of participants' recovery at time of data collection.

### 2.2 Critical Appraisal

Table 1 shows each study's overall quality rating and value for the review rating (see Supplement 3 for a breakdown). Of the 16 studies, nine papers (56.3%) were judged as high quality, and seven papers (43.8%) were judged as medium quality. Consideration of the relationship between the researcher and participants and ethical issues tended to be weaker areas, with a surprising lack of reference to minimizing harm to participants given the nature of the research. Analyses were judged to be insufficiently rigorous for four papers due to lacking clarity on the analytical process (Bell et al. 2024; O'Connell 2023) and concerns around the adequacy of data to support themes (Keski‐Rahkonen and Tozzi 2005; Pilote 1998). Academic theses were generally rated as high quality, with more detailed methods, reflexivity and audit trails, suggesting an inadvertent impact of restricted journal word counts on CASP quality ratings.

With respect to value for the review, eight were judged to be of high or very high value and eight were judged to be of medium value. In papers of higher value, relapse tended to be more directly linked to a study's research question as opposed to an emergent theme examined through its relation to the main research topic such as treatment experiences. Contributing factors to relapse were the most universally explored aspect of experience.

Post‐synthesis sensitivity analyses revealed that the exclusion of all medium quality papers would only result in the loss of two codes (2.1%) and no themes from the synthesis and therefore have an immaterial impact on synthesis findings, but transferability across diagnoses would be impacted. Exclusion of the three papers that contributed to the largest number of codes (Botham 2019; Tibbits 2019; Warchol 2013) would only result in the loss of six codes (6.3%) and no themes, confirming the review was not unduly influenced by a small number of studies.

### 2.3 Thematic Synthesis

The synthesis generated 96 codes which were organized into 14 descriptive themes, before being synthesized into five inter‐related analytical themes (see Supplement S4 for an illustration of the mapping between codes, descriptive and analytical themes). The five themes depicted relapse as 1) enticing (146 coding references), 2) unstoppable (258), 3) protective (243), 4) destructive (182) and 5) instructive (85). Themes span the context, mechanisms and outcomes of relapse, with the first three themes primarily relating to the context of relapse, and the final two focusing more on its outcomes of relapse. All themes address aspects of the mechanisms of relapse.

#### 2.3.1 Theme 1. “I Wasn't Letting Go”: Relapse as Enticing

Summary: Individuals lacked intrinsic motivation for recovery, they remained attached to the disorder and saw appeal in a return to the psychological benefits it provided.

Individuals who relapsed after treatment often attributed this to lacking intrinsic motivation for recovery as typified in the following account:

“I've been in and out of bulimia but I think my problem last time is I didn't – I didn't try very hard. I didn't want it” (adult female BN; Warchol 2013).

Instead, behavioral improvements were commonly achieved through compliance with treatment and external pressure. However, Seed et al. (2016) noted that this external regulation of behavior limited individuals' “investment in change.” They often made plans to relapse after discharge or dropped out of treatment. Once no longer under “surveillance,” they returned to behaviors.

“I knew it wasn't going to work! I was like… I'll humour them and put on the weight, but I know… it won't take me long to take it off again” (adult female, AN; Federici and Kaplan 2008).

Individuals recalled how good the ED made them feel, and expressed doubt about what recovery could offer them. Some saw their ED as something they could do “well,” bringing achievement and esteem. Many held firmly to ideals of thinness which led to a continued valuing of the ED as a means of weight control. In becoming part of individuals' identity, it was even more difficult to give up.

“It was the fear of letting go of that identity … I still really had to know … that bit of me was still there and I wasn't letting go completely” (adult female, AN; Botham 2019).

Some felt inpatient treatment contributed to a sense of needing to do their ED “better,” by exposing them to thinner, “sicker” individuals, new disordered behaviors, and clinical markers of illness that became goal posts to aim for. Individuals also saw how severity elicited care. Therefore, relapse appeared to be a strengthened search for validation in sickness.

“I wasn't convinced that I'd got as bad as I could be … and I needed to prove to myself that I could do it again if I wanted” (adult female, AN; Botham 2019).

Time spent in intensive treatment had also disconnected them from life, separating them from other aspects of their identity and their motivations for recovery. Individuals had become less confident in their ability to cope in normal life, breeding a dependency on the ED and the “bubble” of treatment. When faced with their own perfectionistic expectations in life, relapse provided a “justification” for not meeting these (Seed et al. 2016).

“I had lost all motivation to pursue a career, had no home, no partner, and was accustomed to living in an institution. In these conditions, seeking to do anorexia well (instead of normal life) made sense” (adult female, AN; O'Connell 2023).

For some, treatment had provided a temporary level of accountability and momentum, but their motivation for full recovery waned without support. Some individuals were only “willing to go so far” (Federici and Kaplan 2008) and many held onto certain disordered behaviors, retaining their ED identity. A sense of denial over residual behaviors, such as framing periods of restriction as “intuitive eating” (Liu et al. 2024), allowed them to worsen once more.

#### 2.3.2 Theme 2. “Bound to Lose”: Relapse as Unstoppable

Summary: Treatment had only taken individuals so far; they faced challenges in recovery without support and felt powerless to prevent relapse.

Individuals described the persistence of powerful ED cognitions and associated emotions, beyond physical and behavioral recovery and discharge from treatment. As they acted against their ED, they were subjected to an “overly critical and commanding” internal monologue (Bell et al. 2024) alongside feelings of guilt, self‐disgust and loss of control that felt intolerable. Gaining weight, or even the possibility of this, was a particular trigger, threatening their sense of self, and meaning many “could not psychologically accept it” (Wu and Harrison 2019). As such, intensive weight‐focused models of treatment with minimal psychological support were linked to relapse after discharge.

“Every time a pound goes on you feel absolutely awful you feel really horrible, really fat, really disgusting … it's the feelings that come with it (weight gain) … is what's causing me to relapse so many times” (29‐year‐old female, AN; Bell et al. 2024).

When support was reduced or removed, many individuals lacked confidence that they could continue their recovery. There was a pervasive sense that relapse was all too easy, whereas recovery was effortful and challenging. Their daily lives presented environmental opportunities for relapse that were difficult to resist.

“When you are in hospital … you've got you and a whole team against anorexia … but as soon as you leave hospital and you get home it's just one‐on‐one again and you are bound to lose” (adult female, AN; Stockford et al. 2018).

Individuals had lost the structure of treatment, and some struggled to protect their recovery within their busy schedules. Recovery strategies were forgotten, too difficult to implement in the moment, or discontinued because of overconfidence in their recovery.

“I just feel like doing things on my own is, I don't know (shrugging shoulders). Talking through things, you know, the meetings during the study was helpful then” (adult, BN; Liu et al. 2024).

Individuals struggled to ask for professional support because it was not as readily available and some felt “too recovered” (Cockell et al. 2004). For some, friends and family brought unhelpful diet culture messages and were unsupportive of their recovery.

“I felt really misunderstood by my friends because when I said no to outings … so that I could have my meals … they would be angry” (adult female; Cockell et al. 2004).

Individuals described relapse as the result of intense urges, and obsessions and habit that were difficult to control despite motivation, skills and knowledge.

“I've been very well‐educated about all the health consequences and things like that and I just really didn't want to go down that road but I felt like I couldn't really control it” (24‐year‐old female, BN; Tibbits 2019).

In some sense individuals appeared to lack agency over both their recovery and relapse. Recovery appeared contingent on external support, and relapse seemed out of their hands.

“I thought that maybe … I'd get through it, you know I'd come, I'd come through the illness, … I could actually get better, allow, be allowed to get better, but it wasn't true” (19‐year‐old female, AN; Seed et al. 2016).

One participant who had recovered reflected on the detrimental impact of believing “I don't have a choice, I have an eating disorder” (Botham 2019) and Seed et al. (2016) highlighted the potential for treatment to reinforce this illness perception.

#### 2.3.3 Theme 3. “If the Going Gets Tough I've Always Got This”: Relapse as Protective

Summary: Individuals who faced ongoing psychological difficulties and external stressors and had not learnt alternate ways of coping returned to the comfort and protection of their ED.

Despite their progress in recovery, individuals described underlying psychological difficulties that remained unaddressed. Many referred to poor self‐esteem and difficulties identifying, managing and expressing their emotions. Associated interpersonal challenges were also common, including difficulties with vulnerability and assertiveness, as well as an overconcern with pleasing others. Some felt treatment had not focused enough on these issues or provided healthier coping strategies. Several individuals had substituted the ED with other unhelpful ways of coping such as alcohol. There was a sense that recovery would not be stable and enduring until these underlying difficulties had been addressed.

“I feel like I didn't deal with any of the issues that were there to begin with. The bereavement, the self‐esteem issues … I hadn't dealt with why I had this eating disorder” (adult female, AN; Federici and Kaplan 2008).

These internal vulnerabilities appeared to make external stressors more destabilizing. Individual or cumulative stresses related to work, finances, transitions, physical and mental health, loss, and interpersonal conflict precipitated relapse. Many lacked social support, or their difficulties expressing their feelings and needs prevented them using the support around them.

“We were getting ready to move cross‐country. We were struggling financially … like it all came spiralling at once. And it was too much to handle emotionally and physically and mentally” (32‐year‐old female, BN; Tibbits 2019).

Returning to the ED provided a way to distract or avoid feelings that were difficult to tolerate. It offered a means of control, safety, and comfort, and presented an appealing alternative to relying on others. At the same time, it offered a non‐verbal form of communication of their difficult internal world.

“I relapsed because somebody who had been close in my life for a very long time, finally put an end to it [the relationship], and that created a void for me…my relapse…was to fill something” (20‐year‐old female, BN; De Barbieri 2005).

Individuals' reliance on the ED in these times appeared to be influenced by the knowledge they had previously gained of how it could serve them. Some individuals saw the ED as a “bag of tricks” (Warchol 2013) that helped them to cope or a personified “protector” that would “take over” (Tibbits 2019) when things were difficult. Individuals appeared reassured by the thought that “if the going gets tough I've always got this” (Botham 2019). However, for some, relapse in the face of stressful life events was perceived as inevitable, perhaps because they saw no better alternative.

“Like situations are going to happen and I'm going to react that way. Like with the bulimia in order to feel safe and secure because that's how I feel with it” (adolescent female, BN; Pilote 1998).

For others further in their recovery, Botham (2019) described how it had taken time and multiple relapses to form a recovery that was solid even in difficult times.

#### 2.3.4 Theme 4. “Coming Back With Your Tail Between Your Legs”: Relapse as Destructive

Summary: Individuals felt shame, and feelings of failure in relapse and struggled to be open and accept help.

While relapse could bring positive emotions or apathy, there was also a strong sense of failure, guilt, shame and self‐criticism in relapse. Some individuals criticized their lack of “willpower.” The fact they “couldn't puzzle it together” (Tibbits 2019) despite the knowledge and support they had received, when others could, contributed to a sense there was something wrong with them.

“And like I feel a lot of shame … I just feel like I shouldn't have had to be here four times now … I feel like I failed all the other times” (adult female, BN; Warchol 2013).

Returning to treatment could feel “like coming back with your tail between your legs” (Strand et al. 2017). There was a sense of disappointing others who had invested and supported them in their recovery. This deepened feelings of not wanting to burden others further or being undeserving of additional support. As such, many struggled to speak about and accept help for their relapse.

“Many of the friends I'm thinking of, they had been supportive to me at other points. So with the relapse it was like I don't want to beat a dead horse. I don't want to kind of push up to their limit of being supportive to me” (24‐year‐old female, BN; Tibbits 2019).

Individuals described losing themselves to their ED once more, while outwardly maintaining a façade. They became disconnected from others and neglected themselves. For some, previous negative experiences of treatment contributed to a resistance to approaching support again. Individuals described a need to “look the part” (Cockell et al. 2004) or get even worse before entering treatment.

“Such difficult challenges await at the ward, so the illness wants to take what it can get from you before you go against it” (adult, AN; Strand et al. 2017).

Seeking help often needed to be encouraged by those around them. In contrast, a minority found friends and family unhelpfully ignored or criticized them. Some individuals felt that they were treated in a more restrictive, depersonalized, and judgmental way on re‐entering treatment because of their prior history. Many participants described feeling sad and depressed on relapsing. A sense of losing hope that recovery was possible was common.

“That's one of the most difficult things about being there: that you're sitting there with the same people—myself included— that were there a year ago … and no one has made any progress. It makes you feel a bit hopeless” (adult, AN; Strand et al. 2017).

While individuals feared becoming “one of those revolving door people” (Botham 2019), having experienced a setback, holding hope appeared risky.

“I'm doing really, really well here, but I don't see myself not bingeing and purging when I get home” (adult female BN; Warchol 2013).

#### 2.3.5 Theme 5. “So Much of This Journey … Is Just Learning”: Relapse as Instructive

Summary: Relapse was seen as part of a learning journey; for many it pushed them further in their recovery and did not negate their prior growth.

Some individuals described psychological growth in areas such as self‐esteem, relationships, coping skills, and self‐awareness prior to relapse. While this growth did not prevent them from relapsing, they were not starting again from scratch and there was a sense of cumulative growth in spite of patterns of relapse and recovery. As put by one participant:

“There is a part of me that knows realistically … you've had this chunk of [recovery]… what you learn during that period… not to mention what you learned up to that time, you still … take that with you and keep going and fall back on it” (32‐year‐old female, BN; De Barbieri 2005).

Despite finding themselves in a similar situation, individuals described changes in mindset which affected their response to relapsing. Shifts in emotional reactions to relapse reflected this, such as an increase in disgust responses to purging. Many reported a slow process of learning the destructive impact of the ED and some had built lives in recovery that they wanted to protect. Over time, individuals had grown weary of the ED, such that it was the prospect of relapse and no longer recovery that seemed “just too much work” (Warchol 2013). For some, the relapse provided a turning point pushing them towards seeking support and providing fuel for their recovery.

“When I went into rehab, the second time, I didn't need to be friends with the people in hospital … because I had a life outside to hold on to whereas before, I hadn't built that life yet” (adult female, AN; Botham 2019).

Over multiple relapses, some individuals learnt the importance of seeking early support and responding proactively became easier. For some, they had learnt treatment could provide a “safety net” in case of relapse.

“For me, it has also been useful to ask for help. […] And it gets easier and easier every time. If you've done it once you know how it works” (adult, AN; Strand et al. 2017).

For others, their experiences highlighted the risks of contact with treatment and attempts to avoid further admissions became a motivator for recovery.

“Eventually, I realised that the longer I was in and out of treatment, the further the walls would close in” (adult female, AN; O'Connell 2023).

Relapse also highlighted the work still needed to strengthen their recovery. Individuals accounts showed that through relapse they developed their awareness of triggers and warning signs, learning how relapse could be avoided or identified early.

“An overly rigid food plan is my set up for relapse. I had to find a flexible plan that was right for me before I could become abstinent” (adult female, BN; Wasson 2003).

Many individuals accepted relapse as a part of the journey to recovery and sought to imbue it with purpose. Of note, some did not feel that the term “relapse” captured their experience and instead considered themselves to be in “the process of recovery” (Federici and Kaplan 2008).

“So much of this journey, like yes there's relapse but so much of it is just learning” (27‐year‐old female, BED; Tibbits 2019).

## 3 Discussion

This review synthesized qualitative research on relapse experiences in EDs, drawing on 16 studies and generating five interrelated themes. To the authors' knowledge, it is the first review to specifically focus on this topic. Results highlighted how individuals variously desired, needed or felt powerless to the return of the ED in the face of recovery and life stressors without adequate internal or external resources. Despite supporting individuals to improve behavioral, treatment approaches had fallen short of addressing, or even unwittingly reinforced these vulnerabilities to relapse. Relapse had the potential to bring both feelings of failure and despondency about recovery as well as perspective shifts that motivated and strengthened individuals' recovery.

The interplay between themes mirrored the internal conflict commonly associated with EDs. Individuals experiencing relapse often faced a push‐pull dynamic, feeling both resistant to “letting go” of the disorder and simultaneously “bound to lose” the battle against it. This sense of disempowerment in the face of a perceived inevitability of relapse raises questions for current clinical approaches. In addition, participants' evident insights into why they relapsed hold value for the field. Findings reveal interacting influences of high‐risk situations and ED vulnerability factors which align with elements of models of relapse (e.g., Witkiewitz and Marlatt 2007) and EDs (e.g., Fairburn et al. 2003). For example, persistent body dissatisfaction was linked to relapse both after intensive weight gain and after weight‐neutral treatment (e.g., CBT‐E for BN‐EDs; Liu et al. 2024), in line with existing research on its significance (Keel et al. 2005; McFarlane et al. 2008; Sala et al. 2023). However, the nuances and subjectivity brought out in this review hint at individuals' complex relationships with both the disorder and support systems that are difficult to fully capture within any single model.

The findings, particularly the theme of *relapse as enticing*, underscore the significance of ambivalence in EDs (Colton and Pistrang 2004; Vall and Wade 2015) and emphasize its role in relapse. In line with self‐determination theory (Ryan and Deci 2000, 2017), results indicate that, while treatment may provide extrinsic motivation for recovery, it is intrinsic motivation that is needed to sustain change once support diminishes, and this crucial factor is often lacking.

Findings shed light on the high‐risk period during the lag between individuals' physical and behavioral recovery, and their psychological recovery (Fennig et al. 2017). During this lag, recovery was yet to bring desired benefits and instead confronted individuals with the very issues they had avoided through the disorder. At the same time, the perceived benefits of the ED were all too easy to recall, akin to the pre‐lapse stage in Freeman and Dolan's (2001) model of change.

Through their experiences, individuals had developed an intimate and entangled relationship with the ED which “held” them through life's struggles. This supports literature on the role of identity in EDs and the importance of de‐identification in recovery (Bowlby et al. 2015; Treasure and Schmidt 2013). Despite a focus on motivation and identity within various ED treatments (e.g., MANTRA), the persistence of these issues well into recovery highlighted in the review raise concerns about their underemphasis in relapse prevention work (Schlam and Wilson 2007).

Findings also reveal the influence of social context, including both formal and informal support, on recovery and relapse. In line with previously highlighted iatrogenic impacts (Peebles et al. 2023), inpatient care, for example, was seen as reinforcing thoughts of not being “sick enough,” teaching disordered behaviors, and eliminating recovery motivators, embedding their ED identity.

Individuals presented themselves as powerless against the strength of ED symptoms remaining after treatment, supporting evidence of their association with relapse (McFarlane et al. 2008). This highlights the danger of premature discharges and an inability to provide recommended post‐discharge support (Berends et al. 2018) in the face of high levels of demand on ED services. After discharge, individuals seemed to doubt their ability to manage their own recovery, believing that any improvements were down to treatment rather than their own efforts. Individuals' experiences of medicalized, symptom‐focused models of care appeared to have reinforced a disempowering “sick role,” prompting a sense of victimization and passivity in relapse, as previously noted (Goren‐Watts 2011).

The diversity of experiences conceptualized within relapse is highlighted by this review. The limited cognitive and emotional improvement prior to many experiences labelled as relapse, suggest common behavioral criteria, such as re‐admission, weight change, or an increase in bingeing/purging, mask a persistent dominance of the disorder. The importance of capturing individuals' experiences and how they intersect with ideas of relapse is evident in those who disputed the researcher‐imposed label “relapse.” This review adopts the term “relapse” to connect with existing research. However, it acknowledges that the term carries medical and cultural connotations that may contribute towards the sense of failure and disempowerment identified in findings (DiClemente and Crisafulli 2022).

Findings revealed how individuals', treatment services' and society's interrelated expectations of what illness and recovery “should” look like shaped experiences of relapse (LaMarre and Rice 2021). Individuals felt a sense of failure and shame in non‐linear recovery, hiding struggles or conversely feeling a need to worsen symptoms to fit an expectation of illness. In this way, relapse provides further fuel for the self‐criticism and shame inherent in EDs (Nechita et al. 2021). Concerningly, findings revealed that this internalization of relapse as a personal failure was apparent despite the indications that treatment had failed to meet many individuals' needs.

Following relapse, individuals showed diverse orientations towards recovery, challenging characterizations of relapse as a binary outcome. Mindsets often shifted across multiple relapses, such as gradually de‐identifying with the disorder or experiencing growing demoralization about recovery. Troublingly, relapse could erode trust in oneself, risking dependency on services, and diminish confidence in treatment, deterring help‐seeking. These dynamics show how the psychological impacts of relapse may hinder future recovery efforts.

### 3.1 Strengths and Limitations

This systematic review has several strengths. The breadth of this review enabled relapse to be understood as a complex process rather than a single event examined only through its triggers. Adopting an inclusive conceptualization of relapse allowed for a greater variety of included studies, enriching the review, and fostered a more holistic understanding of relapse which centered individuals' perceptions and personal experience, rather than relying on clinical measures or contact with services to validate their experiences.

Themes were not found to be sensitive to study quality or overly dependent on a small number of studies, supporting the robustness of the findings. The inclusion of academic theses limited the impact of publication bias and contributed thick description from otherwise unheard participants' voices. They also added to the diversity of EDs within the review, important given the dominance of AN research in the field. Exploring relapse from a transdiagnostic lens also enabled the review to capture experiences of diagnostic cross‐over in relapse (e.g., AN to BN; Pilote 1998). This review benefited from including individuals reporting from various stages of recovery, facilitating a diversity of perspectives on their ED (Rossotto et al. 1996). The range of treatments received by individuals suggests the review has captured commonalities in experiences that pose implications for multiple treatment models.

Some limitations were also identified. The exclusion of non‐English language papers can lead to a risk of bias, although only one paper was excluded for this reason. The inclusion of non‐peer‐reviewed theses could be seen to affect quality, though CASP scoring was high. Accounts of relapse experiences that did not constitute a key theme within the paper were excluded but could hold additional value.

Under stricter definitions of relapse, some experiences included in the review might instead be characterized as a lapse (where the setback was brief) or a continuation of the illness (where prior progress was minimal). Defining these experiences as relapse could be viewed as placing undue significance on them. The ability to capture nuances in the experiences of those relapsing after minimal change versus more established recovery was constrained by the lack of contextual information or explicit definition of relapse in included papers.

Transfer of findings should consider that participants were primarily women in Western countries and that experiences noted will likely be shaped by gender and culture (Springmann et al. 2020). Less than half of papers reported participant ethnicity and socioeconomic status, limiting confidence in the transferability of findings.

### 3.2 Future Research

Quantitative research should aim to develop nuanced outcome models that capture the complexities of non‐linear recovery trajectories. Including details on the course of the disorder (e.g., periods of recovery and relapse) and treatment history within participant demographics would provide valuable context beyond ED duration/onset. Additionally, clarity on how relapse has been defined within research is recommended to improve interpretation of findings. Longitudinal studies examining changes in psychological factors post‐treatment could elucidate their interplay and sequencing in relapse, complementing retrospective recall. Further research using discourse analysis to explore how perceptions of relapse are shaped by the language used by individuals and which they encounter in treatment would be a valuable extension to this review. There is a clear gap in research on relapse among males, individuals from the global majority (a term for people who are not considered to be white), and residents of low‐ and middle‐income countries.

### 3.3 Clinical Implications

Overall, the findings have clinical implications for relapse prevention.

- The potential harms of relapse indicate the need for comprehensive support that prioritizes the sustainability of recovery from the outset, with greater emphasis on relapse prevention within treatment.
- Findings caution against a withdrawal of care prior to adequate cognitive and emotional improvements, despite physical and behavioral stabilization. Underlying vulnerabilities (e.g., shape and weight concerns, interpersonal difficulties, and self‐esteem), as well as attitudes towards recovery, particularly intrinsic motivation, self‐efficacy and expectations of recovery, and quality of life beyond the ED emerge as important areas of focus and potential indicators of readiness for discharge.
- Apparent in findings is the need for treatment to validate individuals' ongoing psychological distress in recovery and avoid care models that inadvertently reinforce an escalation of ED behaviors in order to gain support for their distress.
- Findings support a shift towards intensive community treatment as an alternative to inpatient care to reduce unnecessary admissions (NHS England et al. 2019).
- In response to relapse, clinicians should foster hope, self‐compassion, and challenge narratives of failure. Examining individuals' relapse may provide valuable insights for tailoring and guiding treatment.

### 3.4 Public Significance Statement

Relapse is common in eating disorders even after treatment. Treatment and intervention developments have attempted to address this but are limited by our lack of understanding of relapse. The complex nature of relapse is often overlooked in outcome research. This review helps us better understand the experiences of individuals with eating disorders who relapse and offers insights to inform more effective strategies for relapse prevention and intervention.

## Author Contributions

**Natasha Heal‐Cohen:** conceptualization, investigation, methodology, formal analysis, visualization, project administration, writing – original draft, review and editing. **Sophie M. Allan:** conceptualization, supervision, methodology, validation, writing – review and editing. **Nieve Gauvain:** investigation, formal analysis, validation, writing – review and editing. **Rachel Nabirinde:** investigation. **Aaron Burgess:** conceptualization, supervision, project administration, methodology, validation, writing – review and editing.

## Conflicts of Interest

The authors declare no conflicts of interest.

## Supporting information


**Supplementary S1.** Database search strategy.
**Supplement S2.** Papers excluded based on language.
**Supplement S3.** Quality appraisal additional tables.
**Supplement S4.** Illustrations of theme generation.

## Data Availability

Data sharing is not applicable to this review as no new data were created or analyzed. The supplemental file contains the following: database search strategy, non‐English language excluded paper, full quality appraisal table, figures illustrating theme generation.
