# Supplementary material for: Relapse in Eating Disorders: A Systematic Review and Thematic Synthesis of Individuals' Experiences
Source: Clin Psychol Psychother. 2025 Jul 1;32(4):e70101. doi: 10.1002/cpp.70101 (PMC12214294; doi:10.1002/cpp.70101)

Table of Contents

[Supplement S1. Database Search Strategy 2](#_Toc198889434)

[Supplement S2. Papers Excluded Based on Language 3](#_Toc198889435)

[Supplement S3. Quality Appraisal Additional Tables 4](#_Toc198889436)

[Supplement S4. Illustrations of Theme Generation 6](#_Toc198889437)

## Supplement S1. Database Search Strategy

**Table S1**

MEDLINE title, abstract & keyword search strategy

| Search Number | Search string |
| --- | --- |
| #1 | "Eating disorder*" OR anorexi* OR bulimi* OR "binge eating" OR OSFED OR EDNOS |
| #2 | (MM "Feeding and Eating Disorders") OR (MM "Anorexia Nervosa") OR (MM "Binge-Eating Disorder") OR (MM "Bulimia Nervosa") |
| #3 | relaps* OR recur* OR deteriorat* OR worse* OR setback* OR return* OR resum* OR readmission* OR readmit* OR rehospital* |
| #4 | (MH "Recurrence") |
| #5 | "qualitative" OR "mixed method*" OR ethnograph* OR autoethnograph* OR "focus group*" OR "interview*" OR phenomenolog* OR "grounded theory" OR "thematic analysis" OR "interpretive" OR "content analysis" OR "discourse analysis" OR "realist" OR "narrative" OR "textual" |
| #6 | (MH "Qualitative Research+") |
| #7 | #1 OR #2 |
| #8 | #3 OR #4 |
| #9 | #5 OR #6 |
| #10 | #7 AND #8 AND #9 |

## Supplement S2. Papers Excluded Based on Language

1. Cruzat Mandich, C., Díaz Castrillon, F., Kirszman, D., Moncada Arroyo, L., Aspillaga Hesse, C., & Behar Astudillo, R. (2017). Fases de la alianza terapéutica en los trastornos de la conducta alimentaria.

**Abstract:** The aim of this research is to describe the evolution of the therapeutic alliance and its phases in the treatment of patients with eating disorders. A qualitative, analytical-descriptive design was applied based on the Grounded Theory. The sample included 20 Chilean patients suffering from eating disorders according to DSM-5 criteria. Results indicate that therapeutic alliance evolves within three stages that show the following characteristics: 1. First phase (knowledge): distrust, resistance, need for a diagnosis and false complacence predominate; 2. Second phase (trust and consolidation): deeper items are elaborated, therapeutic relationship improves, motivation for treatment emerges; 3. Third phase (re-signification): management of relapses is made, illness is re-defined and the end of the therapy is visualized. A climate of contention, support and security for patients is emphasized, reproducing a comprehensive and unconditional maternal role.

## Supplement S3. Quality Appraisal Additional Tables

**Table S3a**

Individual study ratings against CASP checklist items

| **Value for Review Rating** | Medium | High | High | Medium | Very high | Medium | High | Very high | High | Medium | Medium | Medium | Very High | Medium | High | Medium |
| --- | --- | --- | --- | --- | --- | --- | --- | --- | --- | --- | --- | --- | --- | --- | --- | --- |
| **Overall Quality Rating** | Medium | High | Medium | High | High | Medium | Medium | Medium | Medium | High | High | High | High | High | Medium | High |
| **10. How valuable is the research?** | Yes | Yes | Yes | Yes | Yes | Yes | Yes | Somewhat | Yes | Yes | Yes | Yes | Yes | Yes | Yes | Yes |
| **9. Is there a clear statement of findings?** | Yes | Yes | Somewhat | Yes | Yes | Somewhat | Yes | Yes | Yes | Yes | Yes | Yes | Yes | Yes | Yes | Yes |
| **8. Was the data analysis sufficiently rigorous?** | Somewhat | Yes | Yes | Yes | Yes | Somewhat | Yes | Can't tell | Somewhat | Yes | Yes | Yes | Yes | Yes | Yes | Yes |
| **7. Have ethical issues been taken into consideration?** | Can't tell | Yes | Can't tell | Yes | Can't tell | Somewhat | Can't tell | Yes | Yes | Yes | Somewhat | Somewhat | Yes | Yes | Somewhat | Yes |
| **6. Has the relationship between researcher and participants been adequately considered?** | Can't tell | Yes | Can't tell | Yes | Yes | Somewhat | Can't tell | Yes | Yes | Yes | Yes | Can't tell | Yes | Yes | Somewhat | Yes |
| **5. Was the data collected in a way that addressed the research issue?** | Somewhat | Yes | Somewhat | Yes | Yes | Yes | Yes | Somewhat | Yes | Yes | Yes | Yes | Yes | Yes | Somewhat | Yes |
| **4. Was the recruitment strategy appropriate to the aims of the research?** | Somewhat | Yes | Yes | Yes | Yes | Yes | Yes | Yes | Yes | Yes | Yes | Yes | Yes | Yes | Yes | Somewhat |
| **3. Was the research design appropriate to address the aims of the research?** | Yes | Yes | Yes | Yes | Yes | Somewhat | Yes | Yes | Yes | Yes | Yes | Yes | Yes | Yes | Yes | Yes |
| **2. Is a qualitative methodology appropriate?** | Yes | Yes | Yes | Yes | Yes | Yes | Yes | Yes | Yes | Yes | Yes | Yes | Yes | Yes | Yes | Yes |
| **1. Was there a clear statement of the aims of the research** | Yes | Yes | Yes | Yes | Yes | Yes | Yes | Yes | Yes | Yes | Yes | Yes | Yes | Yes | Yes | Yes |
| **Study** | Bell et al (2024) | Botham (2019) | Cockell et al (2004) | De Barberi (2005) | Federici and Kaplan (2008) | Keski-Rahkonen & Tozzi (2005) | Liu et al (2024) | O'Connell (2023) | Pilote (1998) | Seed et al. (2016) | Stockford et al (2018) | Strand et al (2017) | Tibbits (2019) | Warchol (2012) | Wasson (2003) | Wu & Harrison (2019) |

**Table S3b**

Quality appraisal commentary for example paper: Cockell et al (2004)

| Criteria | Rating | Supporting Comments |
| --- | --- | --- |
| 1. Was there a clear statement of the aims of the research | Yes | Aims explicit alongside their importance and clinical value given risk of relapse soon after discharge |
| 2. Is a qualitative methodology appropriate? | Yes | Justification explicit and appropriate. "The use of qualitative methodology was selected so that a highly detailed account of clients’ phenomenological experiences could be obtained and examined." |
| 3. Was the research design appropriate to address the aims of the research? | Yes | Grounded theory appears appropriate to research aims although not explicitly justified. Justified use of the EDE to assess change in diagnosis and followed up clients at 6 months and why qualitative methodology was used overall. |
| 4. Was the recruitment strategy appropriate to the aims of the research? | Yes | Appropriate and justified as to why following participants who have just finished treatment is best as this is highest period of relapse. Clear who was invited and who ended up participating although no reason given as to why they did not participate. |
| 5. Was the data collected in a way that addressed the research issue? | Somewhat | Some good detail on data collection process, including broad areas for interview questions and how and why this shifted in later interviews to become more specific. More detail on what this looked like could be given and there is no mention of piloting or reviewing the pre-determined questions. The setting for the interviews is also unclear and interview length of half an hour appears short for the broad focus and is not justified. |
| 6. Has the relationship between researcher and participants been adequately considered? | Can't tell | No explicit mention of reflexivity and researchers own subjectivity, assumptions or theoretical stance. |
| 7. Have ethical issues been taken into consideration? | Can't tell | Stated that informed consent was obtained. However, no mention of ethical approval or debrief or limiting harm |
| 8. Was the data analysis sufficiently rigorous? | Yes | Good amount of detail on method. Follows an explicit method. Sufficient data presented to support findings and clear how results are laid out to describe categories and their components with quotes as examples. Also clear how outlying ideas were incorporated. Multiple independent reviewers used but no critical examination of the researcher’s role in the analysis conducted. |
| 9. Is there a clear statement of findings? | Somewhat | Clear findings discussed in relation to research question and literature. However, credibility of findings not discussed. |
| 10. How valuable is the research? | Yes | Clear evidence gap identified, adds to existing findings in a new area (recently discharged patients) and recommendations for practice made as well as suggestions for further research and how these would help with generalisability. |
| Overall Quality | Medium |  |
| Value for the Review | High | Open-ended approach facilitates access to participants' experiences. Significant discussion of factors associated with relapse including signs of relapse (e.g. negative thoughts, reluctance to choose recovery behaviours). Experiences of participants who relapsed sometimes not clearly separated. |

## Supplement S4. Illustrations of Theme Generation

**Figure S4a**

Illustrative example of the process used to map codes to themes for one analytical theme


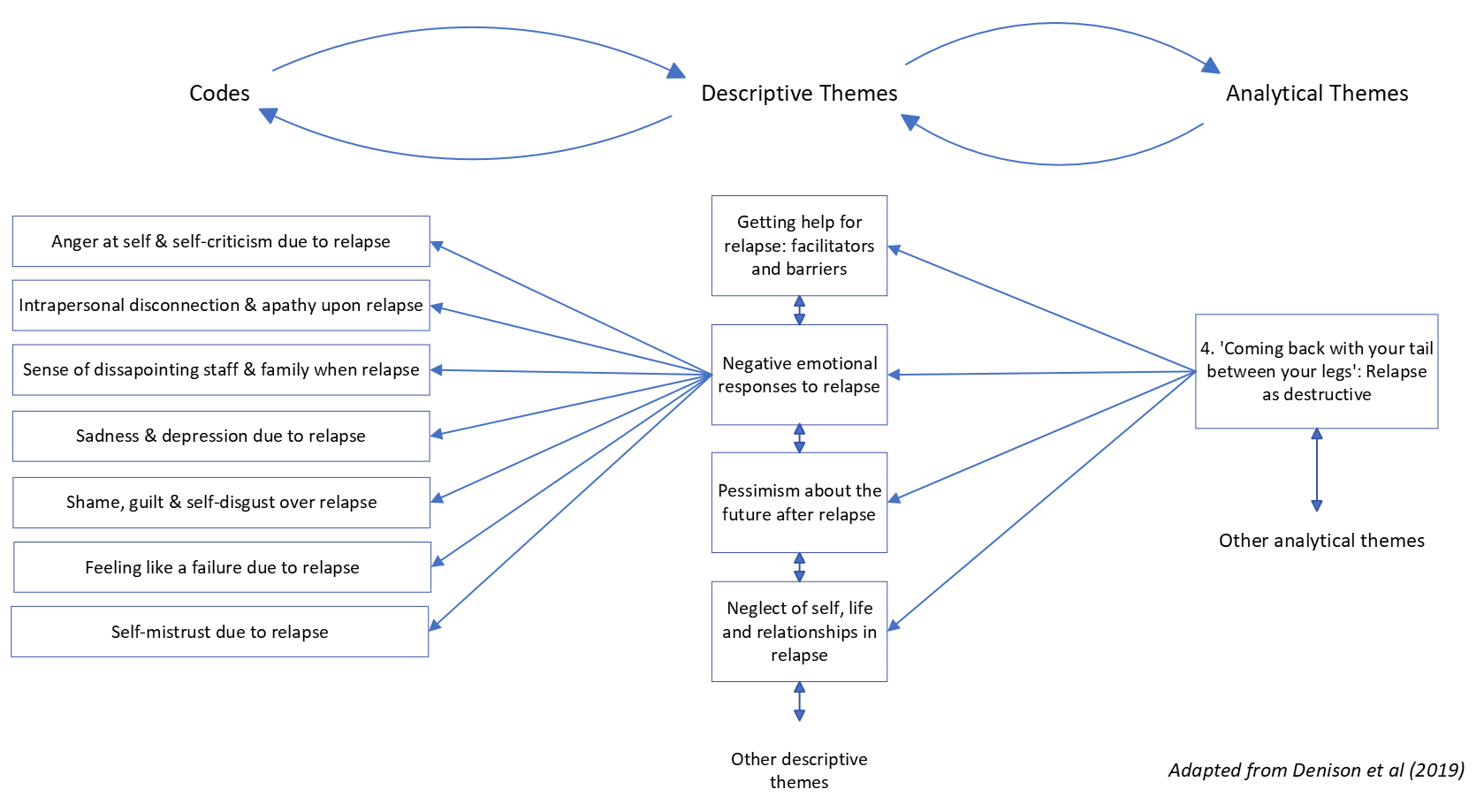


**Figure S4b**

Diagrammatic representation of mapping between descriptive and analytical themes


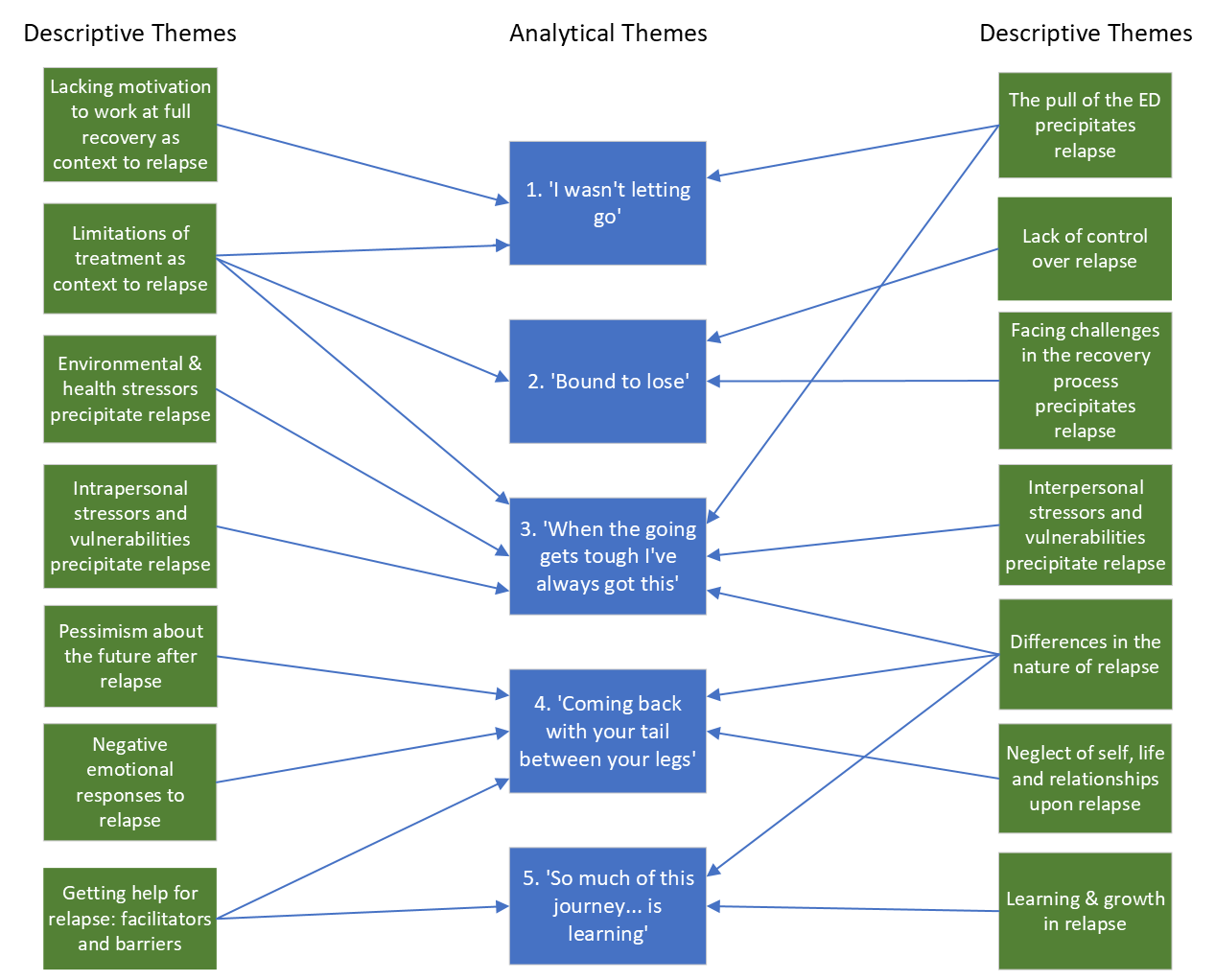

Supplement: Supplementary file 1 — Supplementary S1. Database search strategy. Supplement S2. Papers excluded based on language. Supplement S3. Quality appraisal additional tables. Supplement S4. Illustrations of theme generation. [file CPP-32-e70101-s001.docx]
